# Tumor-derived circulating DNA can induce senescence and SASP activation in mouse embryonic fibroblasts

**DOI:** 10.1007/s10522-026-10400-9

**Published:** 2026-02-05

**Authors:** Ekin Çelik, Ertan Kanbur

**Affiliations:** 1 Department of Medical Biology, Faculty of Medicine, Kırşehir Ahi Evran University, Kırşehir, 40100 Turkey; 2 Department of Immunology, Faculty of Medicine, Kırşehir Ahi Evran University, Kırşehir, 40100 Turkey

**Keywords:** Circulating tumor DNA, cfDNA, Senescence, SASP, DNA damage response, Tumor microenvironment

## Abstract

Circulating tumor DNA (ctDNA), the tumor-originating fraction of cell-free DNA (cfDNA), is widely used as a biomarker for cancer detection and therapeutic monitoring; however, its direct biological impact on normal cells remains insufficiently understood. Since ctDNA contains tumor-derived molecular features, we hypothesized that it could serve as a signal that induces stress responses in healthy stromal cells. In this study, ctDNA and cfDNA were isolated from the conditioned media of B16-F10 melanoma and L929 fibroblast cultures, respectively, and applied to mouse embryonic fibroblasts (MEFs) at concentrations of 100 and 500 ng/mL for 24 h. Senescence was evaluated by SA-β-Gal staining alongside quantitative PCR analysis of senescence and SASP-associated genes, including p16, p21, p53, IL-6, and IL-1β. ctDNA treatment induced a pronounced, dose-dependent increase in senescence marker expression and SASP cytokine production, accompanied by elevated SA-β-Gal staining, whereas cfDNA treatment elicited no significant change compared to controls. These results indicate that ctDNA may act as a biologically active stimulus capable of eliciting senescence-like responses in normal fibroblasts, supporting the possibility that tumor-derived extracellular nucleic acids contribute to alterations in stromal behavior within the tumor microenvironment.

## Introduction

Circulating cell-free DNA (cfDNA) consists of short DNA fragments released into the circulation as a consequence of apoptosis or necrosis (Han & Lo 2021). Elevated cfDNA concentrations have been reported under diverse pathophysiological conditions, including malignancy, trauma, infection, stress, and organ transplantation (Aubert et al. 2024; Hu et al. 2025; Jackson Chornenki et al. 2019; Oellerich et al. 2022). A biologically and clinically important fraction of cfDNA is circulating tumor DNA (ctDNA), which originates from neoplastic cells and recapitulates the mutational profile of the tumor (Arimura et al. 2024; Sassorossi et al. 2025). Although low levels of ctDNA may be detectable in healthy individuals, its tumor-specific molecular content renders it a valuable biomarker for cancer detection, therapeutic monitoring, and genomic characterization.

ctDNA harbors somatic mutations representative of the genetic burden and heterogeneity of both primary and metastatic tumors (Silva et al. 2024). Compared with conventional tissue biopsies, ctDNA analysis offers a minimally invasive strategy that relies on peripheral blood sampling and enables longitudinal assessment of tumor dynamics (Fusco et al. 2025; Pantel & Alix-Panabières 2025; X. Wang et al. 2024a, b, c). Accumulating evidence demonstrates that ctDNA provides critical information on tumor evolution and supports the implementation of personalized therapeutic approaches (Garcia-Murillas et al. 2024; Heider et al. 2021; Merker et al. 2018; Wan et al. 2017).

Despite its diagnostic and prognostic utility, the direct cellular effects of ctDNA remain incompletely defined. Recent studies have indicated that ctDNA may elicit inflammatory responses, influence immune signaling, induce epigenetic alterations, and activate DNA damage pathways (Lee et al. 2024; Mittra et al. 2017). The implications of these processes for normal tissue homeostasis and for the biology of cancer progression remain to be fully elucidated.

Experimental findings further suggest that ctDNA can mediate horizontal DNA transfer, thereby introducing exogenous genetic material into recipient cells and producing measurable biological effects (Cai et al. 2013; Thierry et al. 2016). Tumor-derived DNA has been reported to integrate into the genomes of host cells, potentially initiating oncogenic transformation; however, the mechanisms underlying this phenomenon remain poorly characterized (Abdouh et al. 2019; García-Olmo et al. 2010; Trejo-Becerril et al. 2012).

Cellular senescence is a state in which cells permanently lose their proliferative capacity while maintaining metabolic activity, often referred to as “cellular aging” (Ajoolabady et al. 2025). This process is characterized by an irreversible arrest of the cell cycle in response to various forms of stress or damage (Liu et al. 2025). Principal triggers of senescence include telomere attrition, DNA damage, oxidative stress, and oncogenic signalling (Eppard et al. 2024; Qin et al. 2025). In this context, senescence functions as a protective mechanism at both the cellular and organismal levels, serving to prevent the propagation of deleterious mutations and uncontrolled cell growth (Ungvari et al. 2025; Wright & Shay 2001).

The concept of senescence was first introduced by Hayflick and Moorhead in 1961, who demonstrated that human fibroblasts in culture exhibit a finite replicative lifespan, a phenomenon subsequently termed “replicative aging” (Hayflick & Moorhead 1961). This discovery established a link between senescence and telomere-dependent DNA damage responses (Li et al. 2026; Rattan 2016). Subsequent research has expanded this framework, showing that additional stimuli, including oncogene activation, genotoxic stress, exposure to proinflammatory cytokines, and viral infection, can also induce senescence (McHugh et al. 2024). Beyond its association with organismal aging and age-related diseases, senescence is now recognized as being intricately connected to cancer. Initially regarded as a tumor-suppressive mechanism due to its capacity to halt cell division, accumulating evidence indicates that senescence can also exert tumor-promoting functions through the secretion of a distinct set of bioactive molecules(Yang et al. 2021).

These secreted factors, collectively termed the senescence-associated secretory phenotype (SASP), comprise proinflammatory cytokines, chemokines, growth factors, and proteases (Dong et al. 2024). SASP factors reshape the surrounding tissue microenvironment and have both beneficial and deleterious consequences (Reynolds et al. 2024). In the acute phase, SASP supports immune-mediated clearance of senescent cells and facilitates tissue repair. Conversely, chronic SASP activity sustains a proinflammatory milieu that promotes immune suppression, tissue dysfunction, and tumor progression (Kim et al. 2017; Wang et al. 2020; Yang et al. 2021; Zacarias-Fluck et al. 2015). Indeed, persistent SASP secretion has been shown to enhance cancer cell proliferation, angiogenesis, invasion, and metastatic dissemination (Chambers et al. 2021; Dong et al. 2024; Rostami et al. 2020; Shi et al. 2025). Moreover, the immunomodulatory duality of SASP enables the activation of immune clearance mechanisms while simultaneously facilitating tumor immune evasion through immunosuppressive signaling (Ma et al. 2024).

In addition to endogenous triggers, anticancer therapies such as radiotherapy and genotoxic chemotherapeutics have been shown to induce senescence (Duy et al. 2021). At high doses, these treatments cause cell death; however, at sublethal levels, they promote “therapy-induced senescence,” a phenomenon documented across multiple solid tumors (Prasanna et al. 2021). Clinical and experimental studies have consistently demonstrated upregulation of senescence-associated genes (e.g., p16, p21) and SASP components in patients treated with such agents (Rutecki et al. 2024; Zhang et al. 2025). Notably, therapy-induced senescent cells can secrete factors such as WNT16B, IL-6, and TIMP-1, which attenuate the cytotoxic efficacy of chemotherapeutics on neighboring tumor cells, thereby fostering therapeutic resistance (Sun et al. 2012).

Taken together, these bidirectional effects underscore the complexity of senescence in cancer biology. While senescence provides an essential barrier to malignant transformation, its chronic persistence and SASP-driven signaling may paradoxically contribute to tumor progression and resistance to therapy. Here, we tested whether tumor-derived ctDNA acts as a paracrine signal capable of inducing senescence and SASP activation in normal fibroblasts, thereby contributing to stromal reprogramming within the tumor microenvironment.

## Materials and methods

### Isolation and characterization of cfDNA and ctDNA from cell culture

Murine melanoma B16-F10 cells (Cytion 305,157) and murine fibroblast L929 cells (ATCC CCL-1) were cultured under standard conditions at 37 °C in a humidified atmosphere containing 5% CO_2_. Both cell lines were maintained in DMEM supplemented with 10% fetal bovine serum (FBS) and 1% penicillin–streptomycin. Upon reaching ~ 85% confluence, the culture medium was replaced with serum-free DMEM, and cells were incubated for 24 h to generate conditioned medium (Filatova et al. 2024). Conditioned media were collected and centrifuged at 350 g for 10 min to remove residual cells, and the supernatants were processed for cfDNA isolation. cfDNA and ctDNA were isolated using the ZipPrime cfDNA Isolation Kit (ZipPrime, Turkey), following the manufacturer’s protocol (Çelik et al. 2025). The lysis buffer included in the kit enables recovery of both freely circulating DNA and exosome-associated DNA, thereby ensuring high-yield isolation independent of extracellular compartmentalization.

Quantification of isolated DNA was performed using a Qubit 4 Fluorometer (Thermo Fisher Scientific, USA), and size distribution was analyzed using a 2100 Bioanalyzer microelectrophoresis system (Agilent, USA) (Clausen et al. 2020). All DNA samples were stored at − 20 °C until further use.

### In vitro transfection and acute senescence assay

Mouse embryonic fibroblasts (ATCC CRL-2991) were cultured at 37 °C in a humidified incubator with 5% CO_2_. Upon thawing, cells were expanded, banked, and subsequently used between passages 4–6, a range selected to minimize early post-thaw stress responses and to avoid late-passage senescence-like alterations. For experiments, MEFs were seeded at a density of 1 × 10^5^ cells per well in 6-well plates and maintained until approximately 70% confluence.Transfections were carried out in antibiotic-containing DMEM without FBS using cfDNA or ctDNA isolated at final concentrations of 100 ng/mL or 500 ng/mL for 24 h (Deng et al. 2022; Filatova et al. 2024). For assessment of transfection efficiency, extracellular DNA was subsequently re-isolated from the culture medium, and residual concentrations were quantified with a Qubit Fluorometer (Thermo Fisher Scientific, USA) to determine uptake efficiency (data not shown).

For the positive control, MEFs were treated with 100 nM doxorubicin (DOX) for 24 h following a 24 h incubation under standard conditions (Bientinesi et al. 2022). Negative controls consisted of untreated cells maintained under identical culture conditions. Experimental groups are given in Table 1.

**Table 1:** Experimental groups

Exp. group	Condition	Purpose
NC	Untreated cells	Baseline reference
Sen( +)/PC	100 nM DOX, 24 h	Positive control for senescence
ctDNA-LD	100 ng/mL ctDNA, 24 h	Senescence induction by low-dose ctDNA
cfDNA-LD	100 ng/mL cfDNA, 24 h	Senescence induction by low-dose cfDNA
ctDNA-HD	500 ng/mL ctDNA, 24 h	Senescence induction by high-dose ctDNA
cfDNA-HD	500 ng/mL cfDNA, 24 h	Senescence induction by high-dose cfDNA

### Senescence-associated real-time PCR

A defining feature of senescent cells is permanent cell cycle arrest, mediated by increased expression of cyclin-dependent kinase (CDK) inhibitors such as p16 and p21. In addition, senescent cells secrete a variety of biologically active molecules collectively termed the senescence-associated secretory phenotype (SASP). To capture the heterogeneous nature of senescence, experiments were designed to measure both CDK inhibitors and representative SASP factors at the mRNA level.

Total RNA was isolated and purified using Quick RNA mini-prep RNA isolation kit (Zymo, USA) (Yalman et al. 2020). RNA yield and purity were quantified using a Qubit 4 Fluorometer (Thermo Fisher Scientific, USA). Complementary DNA (cDNA) was synthesized according to the manufacturer’s protocol using the RevertAid First Strand cDNA Synthesis Kit (Thermo Scientific, USA). Quantitative real-time PCR (qRT-PCR) was performed with SYBR™ Green Master Mix, and relative expression levels were calculated using the comparative CT (ΔΔCT) method.

For normalization, β-actin was employed as a conventional housekeeping gene, and PUM1 was additionally included based on its suitability for normalization in senescent cells (González‐Gualda et al. 2021). Primer sequences for the target genes are provided in Table 2.

**Table 2:** Primers used in the study

	F primer (5’-3’)	R primer (5’-3’)	purpose
ACTB	CATTGCTGACAGGATGCAGAAGG	TGCTGGAAGGTGGACAGTGAGG	Housekeeping
PUM1	GCATTTGGACAAGGTCTGGCAG	GCTACAAGTCGAACAGGAGCTC	Senescence housekeeping
p16	TGTTGAGGCTAGAGAGGATCTTG	CGAATCTGCACCGTAGTTGAGC	Senescence
p21	ACGGTGGAACTTTGACTTCGTC	CAGAGTGCAAGACAGCGACAAG	Senescence
p53	TGCTCACCCTGGCTAAAGTT	AATGTCTCCTGGCTCAGAGG	Senescence
IL-6	TACCACTTCACAAGTCGGAGGC	CTGCAAGTGCATCATCGTTGTTC	SASP / Senescence
IL1-β	TGGACCTTCCAGGATGAGGACA	GTTCATCTCGGAGCCTGTAGTG	SASP / Senescence

### Senescence-associated β-galactosidase (SA-β-Gal) activity assay

Senescence-associated β-galactosidase (SA-β-Gal) activity, a hallmark of cellular senescence, was assessed using the Senescence β-Galactosidase Staining Kit (Canvax, USA) according to the manufacturer’s protocol (Takaya & Kishi 2024). Briefly, culture medium was removed, and cells were washed once with 1 × PBS. A 1 × fixative solution was then added, and cells were incubated for 15 min at room temperature. Following fixation, cells were washed twice with 1 × PBS and incubated with 1 mL of SA-β-Gal staining solution per well. Plates were sealed with parafilm to prevent evaporation and incubated overnight at 37 °C in a CO_2_-free incubator. The following day, cells displaying blue staining were imaged under an inverted light microscope at 200 × magnification. Positively stained cells was used as an indicator of senescence.

### Statistical analysis

All experiments were performed in a minimum of three independent biological replicates, with technical replicates included where appropriate. Data are expressed as mean ± standard deviation (SD). Statistical analyses were performed using GraphPad Prism 9.0 software (GraphPad Software, USA). Relative expression levels of senescence markers (p16, p21, p53) and SASP-related genes (IL-6, IL-1β) were calculated using the comparative ΔΔCT method, with β-actin and PUM1 used as internal controls. For comparisons across treatment groups in qRT-PCR, one-way ANOVA followed by Tukey’s multiple comparison test was applied. A p-value < 0.05 was considered statistically significant for all analyses. *p < 0.05, **p < 0.01, ***p < 0.001, ****p < 0.0001.

## Results

### Isolation and characterization of cfDNA and ctDNA from cells

cfDNA and ctDNA were successfully isolated from the conditioned media of L929 fibroblast and B16-F10 melanoma cell cultures, respectively. The isolation protocol yielded highly pure DNA suitable for downstream molecular analyses. Quantification using the Qubit 4 Fluorometer confirmed consistent DNA recovery across replicates, with concentrations within the expected range for culture-derived cfDNA (10–30 ng/mL).

**Fig. 1:**
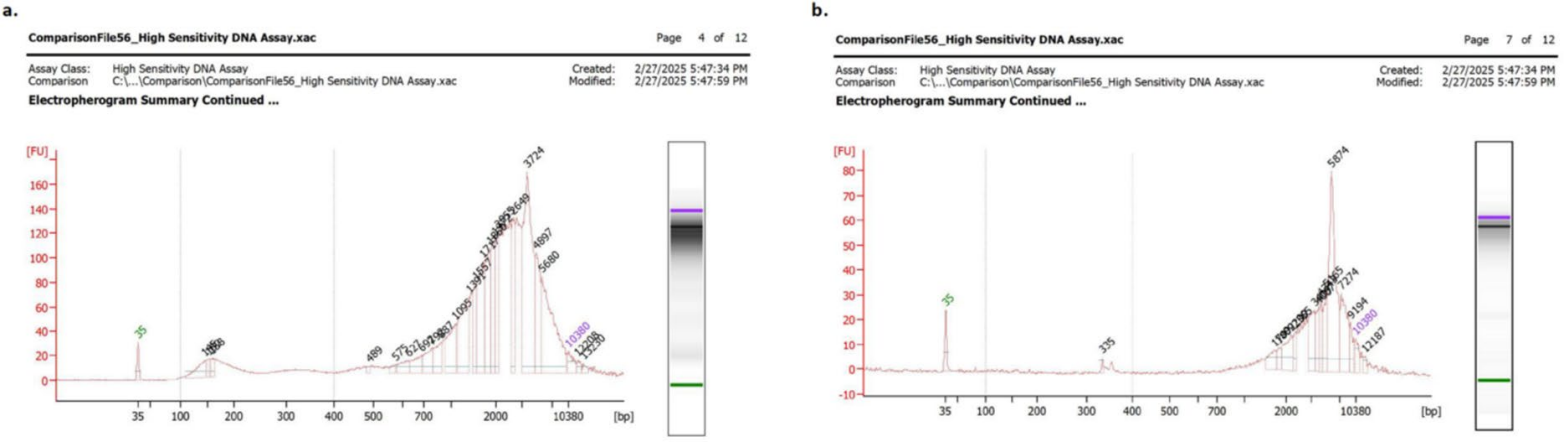
Representative images of microelectrophoresis data of cfDNA (**a**), ctDNA(**b**)

Fragment size distribution profiles obtained using the Agilent 2100 Bioanalyzer revealed characteristic short DNA fragments predominantly between 100–500 base pairs (bp). Cfdna isolated from L929 fibroblast cultures exhibited a slightly broader distribution, with peaks extending up to approximately 500 bp, while ctDNA isolated from B16-F10 cells displayed a more distinct enrichment in shorter fragments below 400 bp, consistent with typical apoptotic DNA fragmentation patterns (Fig. 1).

### Transfection and treatment of MEFs with cfDNA and ctDNA

To evaluate the potential of cfDNA and ctDNA to induce senescence in healthy fibroblasts, mouse embryonic fibroblasts (MEFs) were exposed to low (100 ng/mL) and high (500 ng/mL) concentrations of isolated DNA for 24 h under serum-free conditions. Cell viability and morphology were monitored throughout the treatment period. MEFs treated with ctDNA, particularly at the higher concentration, exhibited mild morphological changes characteristic of early senescence, including increased cell size, cytoplasmic flattening, and a more irregular cell shape compared with untreated controls. In contrast, cfDNA-treated cells largely retained their spindle-shaped fibroblast morphology, similar to the negative control group. These observations suggested that tumor-derived ctDNA may trigger distinct cellular stress responses in normal fibroblasts.

### Gene expression analysis by qRT-PCR

To delineate the molecular profile associated with ctDNA-induced senescence, the expression of cell cycle–regulating genes (p16, p21, p53) and SASP cytokines (IL-6, IL-1β) was quantified by qRT-PCR in MEFs after 24 h exposure to cfDNA or ctDNA at concentrations of 100 ng/mL (low dose, LD) and 500 ng/mL (high dose, HD). Expression levels were normalized to ACTB and PUM1 and analyzed using the 2^ΔΔCt method.

**Fig. 2:**
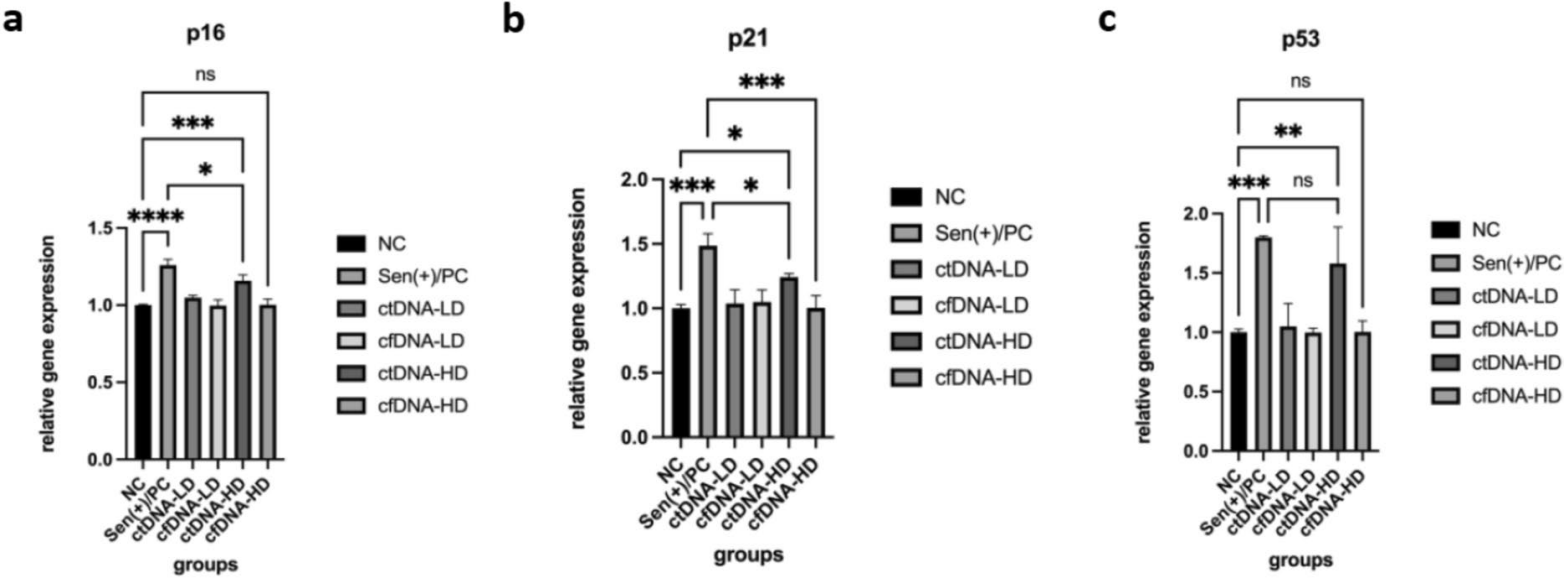
Quantitative analysis of senescence associated gene expression in MEFs following 24 h cfDNA or ctDNA exposure. **a** p16, **b** p21, **c** p53

The results revealed a clear, dose-dependent transcriptional activation of senescence-associated markers in response to ctDNA (Fig. 2a–c). p16 expression increased approximately 1.2 fold (p < 0.001) in ctDNA-HD–treated cells compared with untreated controls, whereas p53 levels rose 1.5-fold (p < 0.01) under the same conditions. In contrast, cfDNA-treated groups showed only mild, statistically non-significant changes (≤ 1.2-fold, p > 0.05) relative to the control. Consistent with these results, p21 expression exhibited a 1.2-fold increase (p < 0.05) following ctDNA-HD exposure, indicating activation of the DNA damage checkpoint pathway.

**Fig. 3:**
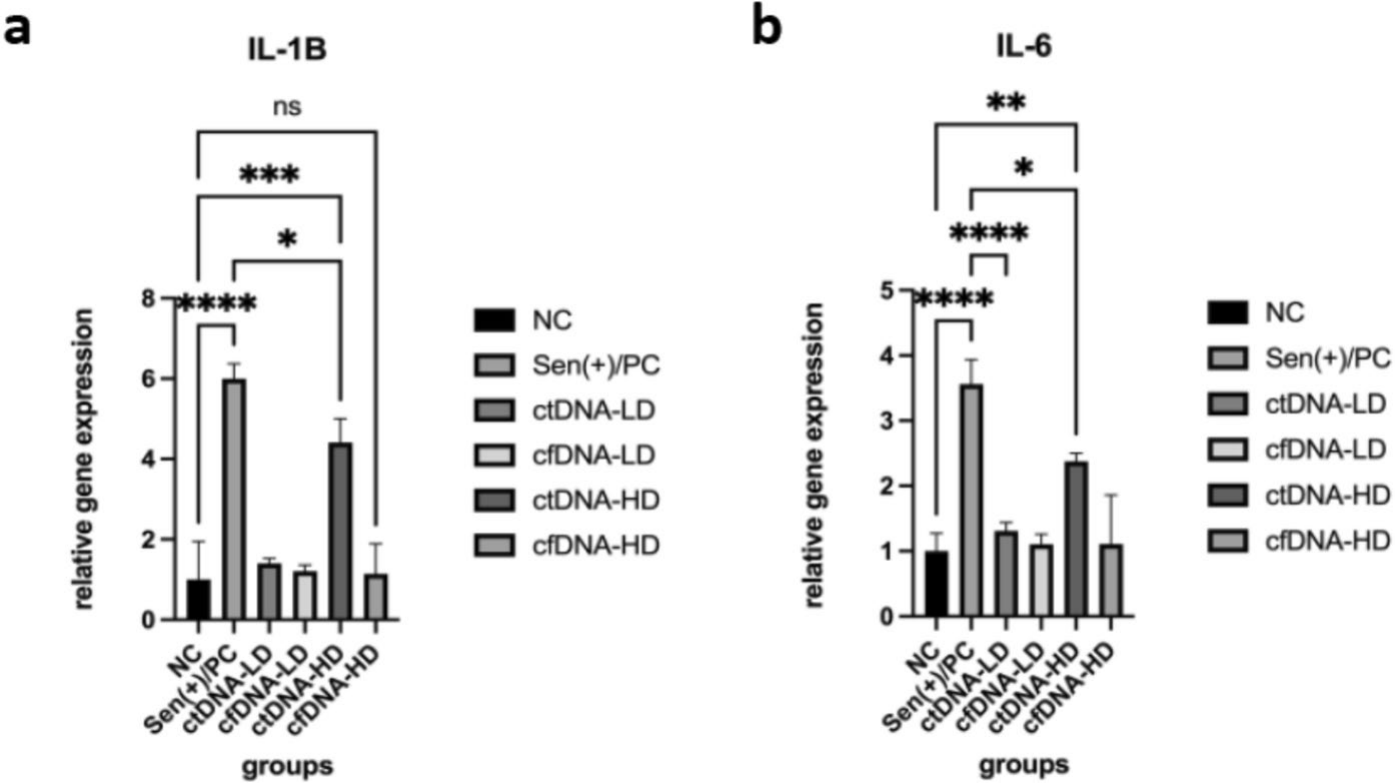
Quantitative analysis of SASP associated gene expression in MEFs following 24 h cfDNA or ctDNA exposure. **a** IL-1B, **b** IL-6

In line with the upregulation of senescence markers, the expression of SASP-related cytokines was markedly elevated following ctDNA exposure (Fig. 3). IL-6 mRNA levels increased 2.4-fold (p < 0.01) and IL-1β increased 4.4-fold (p < 0.001) in the ctDNA-HD group compared to the untreated control, whereas cfDNA-treated cells again displayed no significant alterations (p > 0.05). Importantly, low-dose ctDNA already elicited moderate but statistically not significant increases, supporting a dose-responsive effect.

Collectively, these data demonstrate that ctDNA elicits a robust transcriptional signature characteristic of senescence and SASP activation, engaging the p53/p21/p16 axis and promoting pro-inflammatory cytokine release. The absence of similar responses in cfDNA-treated cells underscores the tumor-derived origin of ctDNA as the determinant of this effect, highlighting its potential role as a paracrine modulator of stromal cell aging and inflammation within the tumor microenvironment.

### Senescence-associated β-galactosidase (SA-β-Gal) staining

**Fig. 4:**
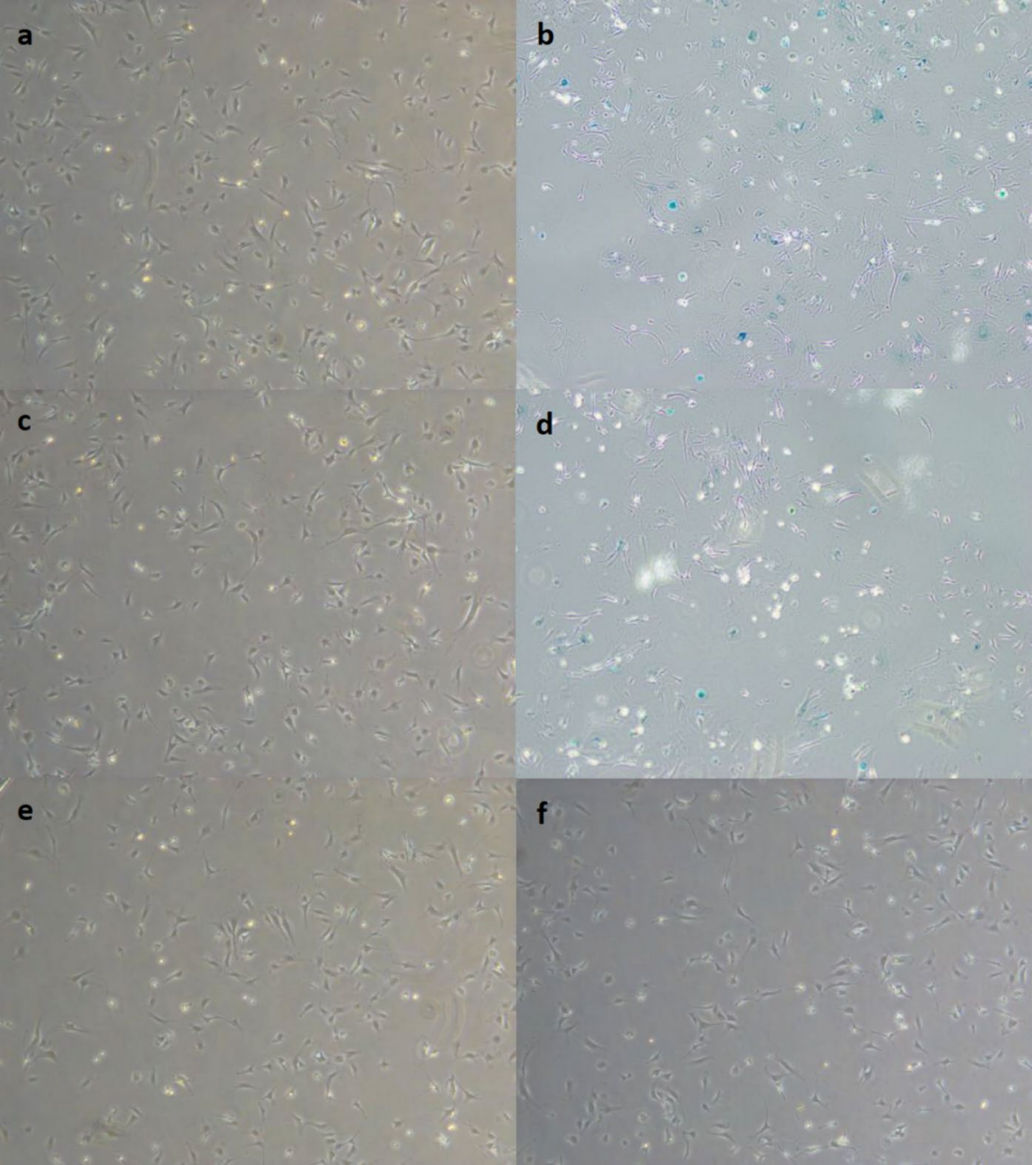
Senescence-associated β-galactosidase staining of MEFs following 24 h exposure to cfDNA or ctDNA. Images captured under brightfield microscopy using a 10 × objective. **a** NC, **b** Sen ( +) /PC, **c** ctDNA-LD, **d** ctDNA-HD, **e** cfDNA-LD, **f** cfDNA-HD

The induction of senescence at the cellular level was further verified using SA-β-Gal staining, a histochemical hallmark of senescent cells. Following incubation with the staining solution, cells treated with ctDNA exhibited a pronounced increase in blue staining, indicative of elevated SA-β-Gal enzymatic activity (Fig. 4). Quantitative evaluation demonstrated that approximately 55–60% of cells in the high-dose ctDNA group were SA-β-Gal-positive, closely resembling the percentage observed in the doxorubicin-treated positive control group (p < 0.01). The low-dose ctDNA group also showed a moderate increase (approximately 20%), whereas cfDNA-treated and untreated control cells exhibited minimal staining (< 20%).

These findings collectively confirm that ctDNA, but not cfDNA, induces a robust senescence phenotype in healthy fibroblasts, characterized by both molecular and enzymatic markers consistent with cellular aging.

## Discussion

To our knowledge, this study provides the first experimental evidence suggesting that tumor-derived circulating DNA may induce senescence in healthy fibroblasts. The findings provide experimental evidence that ctDNA, beyond serving as a diagnostic biomarker, may act as a biologically active molecule capable of modulating the behavior of surrounding normal cells within the tumor microenvironment. The observation that ctDNA treatment leads to upregulation of p16 and p21, two key inhibitors of cyclin-dependent kinases, confirms activation of canonical senescence pathways resulting in irreversible cell cycle arrest. These genes are known effectors of stress-induced premature senescence, typically triggered by DNA damage, oncogenic signaling, or genotoxic stress (Picos et al. 2025; B. Wang et al. 2024a, b, c; Y. Wang et al. 2024a, b, c; Yan et al. 2024).

In addition to cell cycle arrest, ctDNA-treated fibroblasts displayed strong upregulation of SASP-related genes, including IL-6, IL-1. The SASP is a defining feature of senescent cells and exerts profound effects on the tissue microenvironment by promoting chronic inflammation, extracellular matrix remodeling, and angiogenesis (Dong et al. 2024; Kim et al. 2017; Wang et al. 2020). The pronounced increase in these factors observed in the ctDNA-treated groups suggests that ctDNA exposure may not only trigger senescence but also reprogram fibroblasts into a proinflammatory state capable of influencing neighboring cells. Such SASP-mediated alterations could facilitate tumor progression by enhancing cancer cell proliferation, invasion, and immune evasion (Dong et al. 2024; Ma et al. 2024).

The differential response between cfDNA and ctDNA in this study highlights the potential importance of DNA source and composition. ctDNA is known to carry cancer-specific mutations, methylation patterns, and damage-associated molecular motifs that may be recognized by innate immune receptors, such as Toll-like receptors (TLR9), leading to inflammatory and DNA damage responses in recipient cells (Lee et al. 2024). By contrast, cfDNA derived from non-malignant cells lacks these tumor-specific molecular signatures and, therefore, may not activate comparable pathways. This observation supports the hypothesis that the mutational and structural characteristics of ctDNA confer a unique ability to act as a paracrine effector molecule within the tumor microenvironment.

The results of the SA-β-Gal staining assay corroborated the transcriptional findings, demonstrating a clear dose-dependent increase in senescence-associated enzymatic activity following ctDNA treatment. The degree of SA-β-Gal positivity in ctDNA-treated fibroblasts approached the levels observed in doxorubicin-treated positive controls, indicating that ctDNA can elicit a robust senescence-like response comparable in trend to that induced by classical genotoxic agents (Cen et al. 2024; Garcia-Fleitas et al. 2024). These data collectively indicate that ctDNA functions as an exogenous trigger of premature senescence in otherwise healthy fibroblasts.

In addition to inducing senescence, exposure to ctDNA resulted in a partial loss of cell viability, particularly at higher concentrations. This observation suggests that ctDNA can elicit a spectrum of cellular stress responses ranging from reversible growth arrest to irreversible cell death. Mechanistically, this dual outcome is consistent with activation of cytosolic DNA-sensing pathways such as cGAS-STING and TLR9, which detect exogenous DNA and trigger proinflammatory and interferon-stimulated gene cascades (Amadio et al. 2021; Shao et al. 2025; Tao et al. 2024). Sustained activation of these pathways has been shown to induce type I interferon signaling, NF-κB mediated cytokine production, and DNA damage responses that, beyond a certain threshold, shift the balance from senescence toward apoptosis or pyroptosis (Kumar 2021; Liu et al. 2024; Wang et al. 2023). Moreover, excessive accumulation of exogenous DNA fragments can exacerbate genomic instability and reactive oxygen species generation, further amplifying cellular stress. The death of a subset of fibroblasts following ctDNA exposure may therefore represent an adaptive elimination of severely damaged cells, while the surviving population enters a senescent state characterized by p16INK4a and p21, Cip1 upregulation and secretion of SASP factors. This dose-dependent continuum parallels the well-documented phenomenon of therapy-induced senescence, where sublethal genotoxic stress causes growth arrest, whereas higher doses induce apoptotic cell death (Guo et al. 2025; Luo et al. 2025; Saleh et al. 2025). Additionally, experimental conditions such as serum deprivation and antibiotic presence during transfection likely increased cellular sensitivity to ctDNA-induced damage, contributing to the observed cytotoxicity (Chen et al. 2021; Llobet et al. 2015; Zhang et al. 2021).

Physiologically, the coexistence of senescence and cell death could have profound implications for the tumor microenvironment. Dying cells can release additional cfDNA fragments and damage-associated molecular patterns (DAMPs), further activating immune and inflammatory circuits, whereas senescent fibroblasts may perpetuate a chronic inflammatory milieu through SASP secretion (Barkovskaya et al. 2025; Loo et al. 2020). Together, these processes may create a self-reinforcing feedback loop that sustains tissue inflammation and promotes tumor progression. Thus, our findings highlight that ctDNA functions not only as a biomarker of tumor burden but also as a biologically active molecule capable of reprogramming the behavior and fate of surrounding stromal cells.

From a broader perspective, these findings suggest a possible expansion of the biological significance of ctDNA beyond its established diagnostic role in liquid biopsy. If similar effects occur in vivo, ctDNA released from apoptotic or necrotic tumor cells could contribute to the formation of a senescence-enriched, proinflammatory microenvironment that fosters tumor persistence and recurrence. The accumulation of senescent fibroblasts with a SASP-positive phenotype could promote stromal remodeling, enhance vascular permeability, and suppress immune surveillance, thereby facilitating metastatic dissemination. This hypothesis aligns with emerging evidence that therapy-induced senescence can paradoxically promote tumor relapse through SASP-mediated paracrine signaling (Mongiardi et al. 2021; Pacifico et al. 2024; Prasanna et al. 2021).

While this study provides a foundation for understanding ctDNA-induced senescence, several aspects warrant further investigation. The precise molecular mechanisms underlying the recognition and cellular uptake of ctDNA remain to be elucidated. Future studies employing inhibitors of endocytic or TLR9 signaling pathways could clarify the route through which ctDNA exerts its effects. Additionally, extending these findings to in vivo models would help determine whether ctDNA-mediated senescence contributes to systemic inflammatory or metastatic processes.

In conclusion, this study provides preliminary evidence that ctDNA acts as a biologically active entity capable of inducing senescence and SASP activation in normal fibroblasts. These findings suggest a previously unrecognized role of circulating tumor DNA as a signaling molecule that may influence tumor progression through paracrine and microenvironmental remodeling mechanisms. Understanding these interactions could open new avenues for targeting ctDNA-mediated senescence and its downstream inflammatory consequences in cancer therapy.

## Limitations

All experiments were conducted using an in vitro MEF model, and the senescence responses observed may not fully reflect the complexity of stromal-tumor interactions in vivo. The ctDNA examined in this work originated from a single murine melanoma cell line (B16-F10), which may not represent the molecular heterogeneity of ctDNA in human cancers. Although senescence-associated pathways were activated, no mechanistic inhibition experiments, such as TLR9 or cGAS-STING blockade, were performed to identify the upstream sensors responsible for ctDNA recognition. In addition, long-term or irreversible features of senescence were not evaluated beyond the acute 24 h exposure period. Future mechanistic and in vivo studies will be necessary to validate these observations and to clarify the pathways through which ctDNA influences fibroblast function.

## Data Availability

The datasets analyzed during the current study are available from the corresponding author on reasonable request.
